# Safe Laparoscopic Pancreatectomy in a Patient with Rare Superior Mesenteric Artery-Derived Gastroduodenal Artery: A Case Report and Technical Considerations

**DOI:** 10.70352/scrj.cr.25-0415

**Published:** 2025-10-01

**Authors:** Takayuki Miura, Shuichiro Hayashi, Shingo Yoshimachi, Hideaki Sato, Akiko Kusaka, Mitsuhiro Shimura, Shuichi Aoki, Masahiro Iseki, Daisuke Douchi, Shimpei Maeda, Masaharu Ishida, Masamichi Mizuma, Takashi Kamei, Michiaki Unno

**Affiliations:** Department of Surgery, Tohoku University Graduate School of Medicine, Sendai, Miyagi, Japan

**Keywords:** gastroduodenal artery anomaly, superior mesenteric artery, vascular anomalies, minimally invasive surgery, pancreatectomy, multidetector CT

## Abstract

**INTRODUCTION:**

Although several studies have highlighted the importance of recognizing gastroduodenal artery (GDA) anomalies during pancreaticoduodenectomy, their relevance during distal pancreatectomy has not been explored. Herein, we describe the safe performance of laparoscopic distal pancreatectomy in a patient with a rare vascular anomaly, specifically a GDA originating from the superior mesenteric artery (SMA).

**CASE PRESENTATION:**

A 63-year-old woman presented with recurrent pancreatitis due to a cystic lesion in the pancreatic body. Imaging suggested a branch-duct intraductal papillary mucinous neoplasm with substantial ductal stenosis. Multidetector CT (MDCT) revealed a rare anatomical variant: the GDA, arising from the SMA and traversing along the inferior border of the pancreas. Laparoscopic distal pancreatectomy was performed after placing an endoscopic nasopancreatic drainage tube. The aberrant GDA was successfully preserved through careful dissection and vessel loop isolation. Pancreatic transection was completed without vascular injury. A postoperative pancreatic fistula developed and was conservatively managed. Histopathological examination confirmed that the lesion was an intraductal papillary mucinous carcinoma without any invasive features. Follow-up MDCT revealed sustained patency and perfusion of the preserved GDA, right gastroepiploic artery, and anterior superior pancreaticoduodenal artery.

**CONCLUSIONS:**

Laparoscopic pancreatectomy can be safely performed in patients with SMA-derived GDA anomalies, when supported by detailed preoperative imaging and precise intraoperative techniques. These findings highlight the necessity of routine preoperative vascular assessment in patients undergoing minimally invasive pancreatic surgery, reinforcing the broader applicability of these approaches for patients with complicated vascular anatomy.

## Abbreviations

ASPDA
: anterior superior pancreaticoduodenal artery
ENPD
: endoscopic nasopancreatic drainage
ERCP
: endoscopic retrograde cholangiopancreatography
EUS
: endoscopic ultrasound
GDA
: gastroduodenal artery
IPMC
: intraductal papillary mucinous carcinoma
IPMN
: intraductal papillary mucinous neoplasm
MDCT
: multidetector CT
MIPS
: minimally invasive pancreatic surgery
RGEA
: right gastroepiploic artery
SMA
: superior mesenteric artery
SMV
: superior mesenteric vein

## INTRODUCTION

The GDA plays a pivotal role in hepatopancreaticobiliary surgery and exhibits substantial anatomical variation.^1)^ Hepatic arterial variants occur in approximately 20%–50% of individuals^2,3)^; however, anomalies involving the origin of the GDA are relatively uncommon.^4,5)^ Nonetheless, both the origin and branching pattern of the GDA can significantly vary.^1,6)^ Large-scale imaging studies have identified a replaced GDA in approximately 1.1% of cases, with the GDA originating from the SMA in only 0.8% of cases.^7)^

Several studies have emphasized the importance of recognizing GDA anomalies during pancreaticoduodenectomy, particularly in the setting of celiac axis stenosis, where failure to identify these variants may result in hepatic ischemia.^8,9)^ However, their relevance in distal pancreatectomy has received less attention.

The increasing adoption of laparoscopic techniques for pancreatic surgery has introduced specific challenges as surgeons must rely primarily on visual cues for tissue identification and dissection. Accurate preoperative imaging and advanced surgical proficiency are essential for identifying and preserving aberrant hepatic arterial anatomy in operations involving major abdominal structures.^10)^ Preserving anomalous vessels might be necessary for maintaining adequate perfusion to the dependent organs; it requires thorough preoperative planning and intraoperative adaptation.

Herein, we describe a case of laparoscopic distal pancreatectomy for an IPMN in a patient with an anomalous GDA originating from the SMA. This case highlights the value of preoperative MDCT and outlines the technical considerations for safely managing this rare vascular variant.

## CASE PRESENTATION

**Fig. 1:**
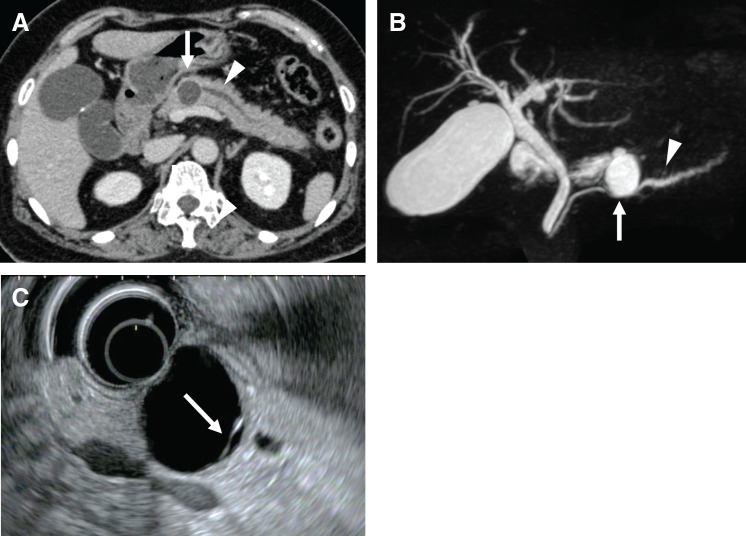
Preoperative imaging. CT axial image (**A**) and MRCP (**B**) show a cystic lesion in the pancreatic body (arrow) and dilatation of the main pancreatic duct at its distal end (arrowhead). Endoscopic ultrasound (**C**) image showing a cystic lesion with internal septations in the pancreatic body (arrow). MRCP, magnetic resonance cholangiopancreatography

A 63-year-old woman was admitted to a local hospital with acute abdominal pain and subsequently diagnosed with acute pancreatitis. She had no significant medical or family history of pancreatic disease. Initial evaluation showed elevated serum pancreatic enzyme levels. CT revealed a 2-cm cystic lesion in the pancreatic body, accompanied by features suggestive of distal obstructive pancreatitis (**Fig. 1A**). The patient was conservatively managed with bowel rest and intravenous fluids, resulting in clinical improvement. However, following oral intake reintroduction, she experienced recurrent abdominal pain and elevated enzyme levels. Approximately 2 weeks after the initial episode, the patient was referred to our institution for further assessment and management. Upon admission, physical examination revealed mild epigastric tenderness without signs of peritonitis. Her vital signs were stable without any evidence of jaundice. Laboratory test results on admission revealed elevated serum pancreatic enzymes (amylase: 476 IU/L; lipase: 1464 IU/L), consistent with acute pancreatitis. Inflammatory markers were not elevated (white blood cell count: 8500/μL; C-reactive protein: 0.53 mg/dL). Tumor marker values prior to surgery were carcinoembryonic antigen, 3.1 ng/mL (normal <5.0) and carbohydrate antigen 19-9, 5.1 U/mL (normal <37), both within normal ranges. Subsequent MRI and EUS revealed a multilocular cystic lesion with internal septations in the pancreatic body (**Fig. 1B** and **1C**). These findings were more consistent with a branch-duct IPMN or another neoplastic cystic lesion rather than a pseudocyst. ERCP revealed extrinsic compression and stenosis of the main pancreatic duct at the lesion site. Attempts to obtain ERCP brush cytology were unsuccessful due to the difficulty in accessing the narrowed duct. Given the presence of high-grade stenosis and risk of recurrent pancreatitis with oral intake, the patient was kept nil per os, and an ENPD tube was placed in preparation for surgical intervention. Pancreatic juice cytology was obtained through the ENPD tube. The specimen revealed thin mucin and sheet-like clusters of columnar epithelial cells with moderate atypia. Some cell clusters exhibited cytoplasmic mucin accumulation, consistent with features of IPMN.

**Fig. 2:**
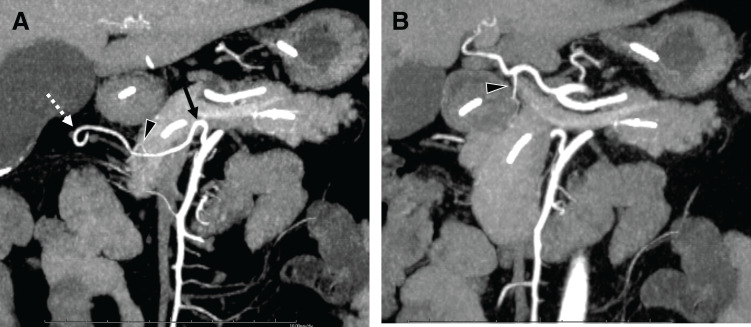
Preoperative multidetector CT. (**A**) Coronal CT image of the abdomen shows the GDA (arrow) originating directly from the SMA. The GDA gives rise to the RGEA (dotted arrow) and ASPDA (arrowhead). (**B**) The PSPDA (arrowhead) arising from the CHA, is visible as a very thin vessel. ASPDA, anterior superior pancreaticoduodenal artery; CHA, common hepatic artery; GDA, gastroduodenal artery; PSPDA, posterior superior pancreaticoduodenal artery; RGEA, right gastroepiploic artery; SMA, superior mesenteric artery

**Fig. 3:**
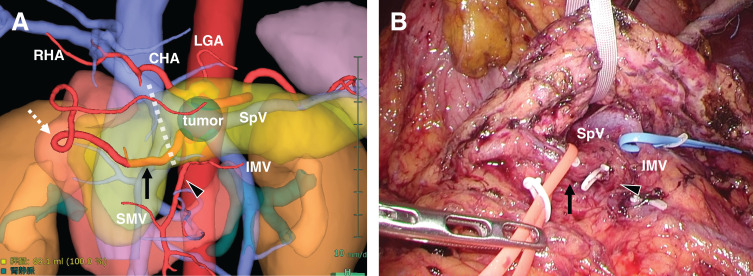
Preoperative imaging and intraoperative findings. (**A**) Preoperative 3D reconstruction CT image (created using Synapse Vincent, Fujifilm, Tokyo, Japan) showing the GDA (arrow) arising from the SMA (arrowhead). The GDA courses inferior to the pancreas at the planned transection site and gives rise to the RGEA (dotted arrow). The planned pancreatic transection line (dashed marker). (**B**) Intraoperative photograph showing the GDA (arrow) arising from the SMA (arrowhead). A vessel tape was placed around the GDA, and a cotton tape was used to retract the pancreatic body, facilitating safe dissection of the aberrant artery. CHA, common hepatic artery; GDA, gastroduodenal artery; IMV, inferior mesenteric vein; RHA, right hepatic artery; SMA, superior mesenteric artery; SMV, superior mesenteric vein; RGEA, right gastroepiploic artery; SpV, splenic vein

The indication for surgery was based on multiple clinical and radiological factors. The patient had experienced recurrent episodes of obstructive pancreatitis, with elevated pancreatic enzymes and pain recurring upon oral intake. Imaging revealed a multilocular cystic lesion with significant stenosis of the main pancreatic duct, suggesting a branch-duct IPMN. Although cytological malignant confirmation could not be obtained, the persistent ductal obstruction, clinical deterioration, and failure of conservative management raised concerns about neoplastic progression. We decided to proceed with surgery to prevent further episodes of pancreatitis, obtain a definitive histopathological diagnosis, and exclude any possibility of malignancy. Preoperative MDCT was used for surgical planning. It identified a rare vascular anomaly in which the GDA originated from the SMA rather than its typical origin from the common hepatic artery. The GDA continued as the RGEA and ASPDA (**Fig. 2**). The anomalous GDA arose ventrally from the SMA and ran along the inferior border of the pancreas, close to the planned transection line (**Fig. 3A**).

Intraoperatively, the GDA was found to originate from the ventral aspect of the SMA, running horizontally along the inferior border of the pancreas and anterior to the SMV, consistent with the preoperative imaging. Early taping of the inferior mesenteric vein allowed visualization of the splenic vein–portal vein confluence from the inferior aspect of the pancreas. The surgeon repositioned to the patient’s left side and performed isolation of the GDA arising from the SMA via the left-sided inferior border of the pancreas. This maneuver enabled safe taping of the pancreatic body without compromising the aberrant GDA. Intraoperative ultrasound was not used to detect the aberrant GDA, as preoperative MDCT provided sufficient anatomical clarity, and intraoperative dissection proceeded without difficulty in vessel identification (**Fig. 3B**). The pancreas was transected using a surgical stapler to preserve the anomalous artery. Intraoperative frozen-section analysis of the pancreatic margin confirmed the absence of malignancy, thereby ensuring adequate oncological resection. The procedure was completed without compromising the arterial perfusion.

The patient developed a postoperative pancreatic fistula, which was classified as Grade B according to the international study group on pancreatic fistula.^11)^ The condition was conservatively managed with drainage and close monitoring. No additional intervention was required; the patient improved and was discharged after fistula resolution. Histopathological examination confirmed the diagnosis of IPMC without any evidence of invasion. A limited regional lymph node dissection was performed as part of the oncological resection. A total of 7 lymph nodes were retrieved and examined. No lymph node metastases were identified on histopathological evaluation, consistent with a diagnosis of non-invasive IPMC. Follow-up contrast-enhanced CT demonstrated sustained patency of the preserved anomalous arteries, including the GDA originating from the SMA, RGEA, and ASPDA. The patient remained asymptomatic at follow-up, without evidence of disease recurrence.

## DISCUSSION

This case report highlights 2 important considerations for managing pancreatic neoplasms in patients with an aberrant vascular anatomy. First, preoperative MDCT is essential for identifying rare anatomical variants and optimal surgical planning to prevent postoperative complications related to compromised perfusion of the stomach and adjacent organs. Second, it demonstrates that laparoscopic distal pancreatectomy can be safely performed in patients with a GDA originating from the SMA, provided appropriate preoperative evaluation and careful intraoperative dissection are performed. These findings broaden the scope of vascular anomaly management in hepatopancreaticobiliary surgery beyond pancreaticoduodenectomy and emphasize the importance of comprehensive vascular mapping in MIPS.

**Fig. 4:**
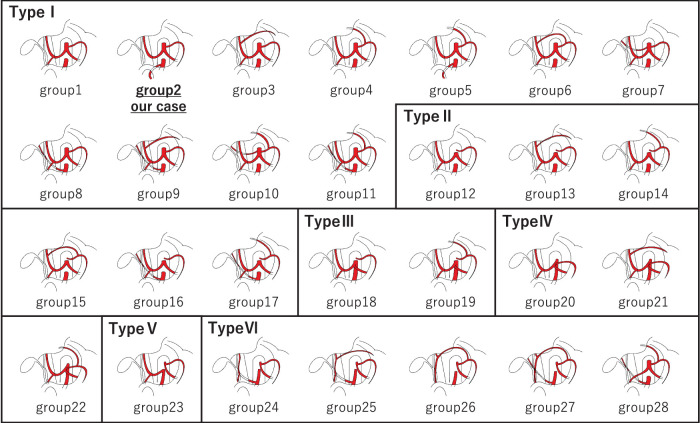
Schematic diagram of Adachi’s celiac and superior mesenteric artery classifications.^12)^ The figure illustrates representative types from Adachi’s classification, which describes anatomical variations in the origins of major abdominal arteries based on cadaveric studies. Type I: The celiac trunk gives rise to the LGA, CHA, and SA. The SMA arises independently from the aorta. This is the most common configuration. Type I is further subclassified depending on the presence of accessory hepatic or left gastric arteries. Type II: The LGA arises directly from the aorta, while the CHA and SA originate from the celiac trunk. Type III: The CHA, SA, and SMA arise from a common trunk, and the LGA arises separately from the aorta. Type IV: The celiac trunk and SMA arise from a common trunk (celiacomesenteric trunk). Type V: The LGA and SA form a common trunk, while the CHA and SMA arise together from a separate trunk. Type VI: The CHA is absent, while the LGA and SA arise from a common trunk. CHA, common hepatic artery; LGA, left gastric artery; SA, splenic artery; SMA, superior mesenteric artery

**Fig. 5:**
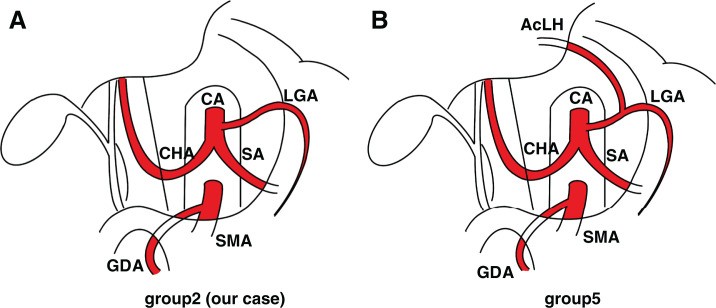
Variants of Adachi Type I classification illustrating GDA branching from the SMA.^12)^ (**A**) Type I group 2–Present case: The celiac trunk gives rise to the LGA, CHA, and SA. The SMA independently arises from the aorta and gives off an anomalous GDA. (**B**) Type I group 5–With aLHA: In this variation, the GDA also originates from the SMA, but aLHA arises from the LGA. The CHA still originates from the celiac trunk. aLHA, accessory left hepatic artery; CHA, common hepatic artery; GDA, gastroduodenal artery; LGA, left gastric artery; SA, splenic artery; SMA, superior mesenteric artery

Our patient exhibited a rare arterial variant consistent with the Adachi Type I group 2 classification, in which the GDA arises from the SMA and continues as the RGEA.^12)^ This configuration differs from the standard anatomy, where the GDA typically branches from the common hepatic artery. Adachi’s classification, developed initially through cadaveric studies, categorizes the branching patterns of the celiac and SMAs into 6 major types with subgroups (**Fig. 4**). This variant of the present case is observed in only 2.4% of adults, underscoring the importance of accurate preoperative identification, which is frequently overlooked.^5)^ Additionally, 2 subtypes of Adachi Type I, both featuring the GDA originating from the SMA, are a rare but surgically relevant anatomical variation (**Fig. 5**). Understanding this classification helps anticipate unusual vessel courses that may otherwise be overlooked during hepatopancreaticobiliary surgery. The prevalence and configuration of such anomalies vary across populations. MDCT-based studies have described various patterns, including a replaced GDA coexisting with replaced or accessory hepatic arteries.^13)^ To the best of our knowledge, there are very few, if any, published reports describing the preservation of an SMA-derived GDA during distal pancreatectomy. Most existing literature focuses on this vascular variant in the context of pancreaticoduodenectomy due to its implications in hepatic arterial perfusion.^10,14)^ This makes the present case particularly notable, as it illustrates the relevance of this vascular anomaly even in distal pancreatectomy and emphasizes the importance of considering such variants regardless of resection type.

Although surgical attention during distal pancreatectomy has traditionally focused on the splenic vessels, there is a growing emphasis on detailed vascular mapping of the hepatic and mesenteric vasculature, even in open procedures. With the increasing availability of high-resolution CT, preoperative vascular evaluation is becoming a standard part of surgical planning in both open and MIPS.^15)^ This case reinforces the need for such routine assessment, particularly for identifying rare arterial variants that may affect surgical safety and organ perfusion. Failure to recognize aberrant anatomy intraoperatively may result in unintentional vascular injury, leading to hemorrhage, ischemia, or technical difficulties during resection. Therefore, a good understanding of embryological development and vascular variations is critical.^16)^ Previous studies have supported the use of high-resolution imaging modalities, such as CT or MRI, to guide safe dissection in patients with a complex vascular anatomy.^17,18)^ The significance of our findings is further supported by reports indicating that rare coexisting vascular variants can present surgical challenges, leading to unintended vessel injury when a comprehensive preoperative evaluation is lacking.^19)^ Our case reinforces these observations, demonstrating that high-quality MDCT enables predicting vascular complexity and facilitates establishing a precise surgical strategy, ultimately contributing to safe and favorable outcomes.

Laparoscopic distal pancreatectomy has become the standard approach for treating benign or malignant lesions of the pancreatic body and tail, as it offers reduced morbidity and faster recovery than open surgery.^20,21)^ Identifying and preserving the aberrant GDA originating from the SMA was a key technical challenge in this case. Aberrant arteries often course through or along the pancreatic head or body, placing them at risk of injury during dissection. In our patient, the GDA ran horizontally along the inferior border of the pancreas, anterior to the SMV, directly in line with the intended transection site. This anatomical configuration made preservation particularly difficult. Preoperative imaging enabled precise localization, and the vessel loops provided gentle traction intraoperatively, which facilitated safe identification and dissection. This technique allowed separation of the pancreatic parenchyma from the artery, maintaining vascular integrity and minimizing the risk of hemorrhage or ischemia.^22)^ However, complex dissection to preserve the vessels may increase the risk of pancreatic injury and fistula formation. This case illustrates that laparoscopic pancreatectomy can be safely performed in patients with a complex vascular anatomy, who might otherwise have been considered as candidates for open surgery.

The decision to perform laparoscopic rather than robot-assisted distal pancreatectomy was made based on institutional protocols and surgeon expertise. At our center, both laparoscopic and robotic approaches are utilized for MIPS, with the choice depending on tumor location, vascular involvement, patient factors, and resource availability. In this case, the surgical team had extensive experience in advanced laparoscopic pancreatic procedures. Moreover, the vascular anatomy, while complex, was deemed manageable without robotic articulation. Additionally, robot-assisted pancreatic surgery was in its early phase of implementation at our institution at the time; laparoscopic resection was considered more appropriate based on the available resources and team familiarity. The indication for MIPS at our institution includes benign and malignant tumors without major vascular invasion or infiltration to adjacent organs, provided that safe resection can be ensured through preoperative imaging and intraoperative assessment.

Clinically, preserving anomalous vessels such as the GDA originating from the SMA is essential for preventing postoperative ischemic complications and pseudoaneurysm formation at the arterial stump caused by pancreatic fistula.^23)^ Injury to these arteries can compromise the blood flow to the stomach and duodenum, potentially resulting in delayed gastric emptying, gastroduodenal ulceration, or acute pancreatitis.^24)^ Even ligation of the accessory arteries during pancreatic surgery may lead to ischemia due to limited collateral circulation.^25)^ In our case, postoperative imaging confirmed the patency of the preserved vessels, including the RGEA and ASPDA, which originated from the aberrant GDA. These findings support the clinical importance and technical feasibility of vessel preservation during MIPS. Although GDA anomalies have been well-documented in the context of pancreaticoduodenectomy,^10,17,18)^ their relevance in distal pancreatectomy has been less thoroughly explored. This case addresses this gap by demonstrating that such vascular anomalies can have significant surgical implications, even during distal pancreatectomy. Advances in high-resolution CT with 3D reconstruction now allow for accurate preoperative identification of these variants, enabling safe laparoscopic management and expanding the eligibility for MIPS.

Although the hepatic arterial anatomy was normal in this case, injury to the aberrant GDA could have potentially compromised the arterial inflow to the ASPDA and RGEA, increasing the risk of localized gastric or duodenal ischemia. In the event of GDA injury, intraoperative assessment of tissue perfusion—including visual inspection and indocyanine green fluorescence imaging, if necessary, has guided the decision of whether arterial reconstruction was required. Specific findings such as pallor of the gastric or duodenal wall, loss of color Doppler signal in the ASPDA or RGEA, or delayed capillary refill would have prompted consideration of vascular reconstruction or conversion to open surgery.

Importantly, the implications of this case extend beyond a single successful procedure. As minimally invasive techniques become increasingly prevalent in pancreatic surgery, a thorough preoperative vascular assessment becomes more critical. Failure to detect vascular anomalies in advance may result in prolonged operative times, increased blood loss, conversion to open surgery, or ischemic complications, such as pancreatic necrosis. Our findings reinforce the importance of detailed imaging and preoperative planning to minimize these risks and ensure safe surgical outcomes. Although the use of pancreatic protocol CT and vascular mapping is already standard practice in high-volume pancreatic centers, the value of this case lies in how preoperative imaging enabled the identification of a rare SMA-derived GDA variant that significantly influenced the surgical strategy. The detailed vascular assessment was not only confirmatory but also directly contributed to selecting the laparoscopic approach and guiding dissection to avoid vascular injury. Thus, this case exemplifies how high-resolution preoperative imaging can translate into safer and more precise technical execution in complex vascular scenarios.

## CONCLUSIONS

This case demonstrates that laparoscopic pancreatic resection can be safely performed in patients with rare vascular anomalies, such as a GDA originating from the SMA, provided thorough preoperative imaging and planning are undertaken. Careful vascular mapping and intraoperative technique enabled the successful identification and preservation of this rare variant in the present case, preventing ischemic complications and resulting in favorable surgical and oncological outcomes. These findings underscore the importance of routine preoperative vascular assessment in patients undergoing MIPS and support the broader applicability of laparoscopic approaches in those with complex vascular anatomy.

## DECLARATIONS

### Availability of data and materials

No datasets were generated or analyzed during the current study.
